# Identifying Clinical Measures Related to Falls in Ambulatory Patients with Spinal and Bulbar Muscular Atrophy

**DOI:** 10.3390/neurolint17060080

**Published:** 2025-05-23

**Authors:** Joseph A. Shrader, Allison C. Niemic, Rafael Jiménez-Silva, Joshua G. Woolstenhulme, Galen O. Joe, Uma Jacobs, Ashwini Sansare, Angela Kokkinis, Kenneth Fischbeck, Chris Grunseich, Cris Zampieri

**Affiliations:** 1 Rehabilitation Medicine Department, Clinical Center, National Institutes of Health, Bethesda, MD 20892, USA; 2 Neurogenetics Branch, National Institute of Neurological Disorders and Stroke, National Institutes of Health, Bethesda, MD 20892, USAchristopher.grunseich@nih.gov (C.G.)

**Keywords:** Kennedy’s disease, Timed Up and Go, Adult Myopathy Assessment Tool, falls, balance, fixed-frame dynamometry, plantarflexor strength, mobility

## Abstract

Introduction: Spinal and bulbar muscular atrophy (SBMA) is an adult-onset, X-linked, progressive neuromuscular disease caused by abnormal CAG trinucleotide expansion in the androgen receptor gene. Patients with SBMA report difficulty with falls on self-reported activities of daily living scales. To our knowledge, no study has examined the relationship between falls and common clinical measures of strength, balance, mobility, and disease biomarkers. We performed a cross-sectional analysis of an SBMA cohort. Objectives: The objectives of this study are as follows: (1) compare demographics, clinical measures, and biomarkers between patients who did and did not fall; (2) determine which measures best discriminate fallers from non-fallers; and (3) identify cutoff scores to detect patients with a higher fall risk. Design: Cross-sectional analysis was used. Outcome Measures: Disease biomarkers included blood serum creatinine, and clinical measures included the Timed Up and Go (TUG), the Adult Myopathy Assessment Tool (AMAT), and posturography, including the Modified Clinical Test of Sensory Interaction on Balance and the Motor Control Test. The Maximal Voluntary Isometric Contractions (MVICs) of four lower extremity muscles were captured via fixed-frame dynamometry. Results: We identified three clinical measures that help detect fall risk in people with SBMA. A post hoc receiver operating characteristic curve analysis helped identify cut scores for each test. Impairments of mobility (TUG > 8 s), muscle endurance (AMAT endurance subscale < 14), and muscle strength (ankle plantar flexion MVIC < 45% of predicted) were different between fallers and non-fallers, via independent *t*-tests. Conclusions: These three clinical tests can help detect fall risk that may help clinicians implement gait aid use or other fall prevention strategies before catastrophic falls occur.

## 1. Introduction

Spinal and bulbar muscular atrophy (SBMA) is an adult-onset, X-linked, progressive neuromuscular disease [1] caused by abnormal CAG trinucleotide expansion in the androgen receptor gene, affecting males only [2]. Research suggests that dysfunction is due to both neurogenic [3] and myogenic [4,5] pathologies, with motor neuron and skeletal muscle changes contributing to the hallmark clinical characteristics of progressive bulbar and extremity muscle weakness. Additional musculoskeletal symptoms include muscle fasciculations, tremors, cramping, fatigability, dysarthria, and dysphagia [6]. Patients also experience sensory nerve abnormalities [7,8] and signs of androgen insensitivity [9].

Progressive loss of muscle strength, while relatively slow in SBMA (average of 2% decrease in maximum strength per year), leads to profound deficits in functional ability over time [10]. In the largest cohort to date, the median age of muscle weakness onset was 44 years, the median age of cane use was 59 years, and the median age of wheelchair use was 61 years [11]. Patients most frequently reported mobility impairment and muscle weakness as the most disabling disease-related issues [12]. Eighty-four percent of ambulatory individuals with SBMA reported having problems with falling on a self-report scale [13], and 64% of an SBMA control group in the placebo arm of a treatment trial reported a fall during the 2-year study period [10], with both studies being part of the same cohort. In a recent study, seven out of seven individuals with SBMA reported a fall during the previous year [14].

In a systematic review with a combined sample size of >6000 community-dwelling (eight studies) and institutionalized older adults (six studies), age 60 and above, the five biggest risk factors for falls were muscle weakness, history of falls, gait deficits, balance deficits, and necessity of gait aid usage [15]. Men with SBMA can often present with all five of these risk factors, in addition to sensory neuropathy, putting them at high risk for falling. Falls often lead to injuries, such as broken bones or head injuries, fear of falling, inactivity, loss of independence, and hospitalizations [16]. In similar neuromuscular disorders where falls are a concern, such as Amyotrophic Lateral Sclerosis (ALS), lower extremity muscle strength and functional mobility tests [17] have been found to be helpful in determining which patients were likely to fall. To our knowledge, there have been no investigations to identify factors that predispose individuals with SBMA to falling.

We performed a cross-sectional analysis of an SBMA cohort to (1) compare demographics, clinical measures, and biomarkers between patients who did and did not fall; (2) determine which factors best discriminate fallers from non-fallers; and (3) identify cutoff scores to detect patients with a higher fall risk.

## 2. Materials and Methods

### 2.1. Participants

Fifty males (mean age 55.0 ± 9.0 years, range 29–74 years, disease duration 15.6 years ± 9.2 years) included in this cross-sectional analysis were initially recruited for participation in a larger randomized controlled trial investigating the effects of 12 weeks of functional exercise versus stretching (control group) for individuals with SBMA (NCT01369901) [18]. Inclusion criteria required that participants be ambulatory, over 18 years of age, diagnosed with SBMA as confirmed by genetic testing, and scored between 14 and 41 on the Adult Myopathy Assessment Tool (AMAT) [19]. The trial protocol was approved by the NIH Institutional Review Board, and informed consent was obtained from all the participants. The analysis includes only data from their baseline visit before the exercise regimen had been initiated. Gait aid use was determined at the initial visit according to the question, “Do you typically use a gait aid?” Patients’ scores were compared to reference values provided by the manufacturer of the balance testing system (SMART Equitest^®^ System by NeuroCom; previously Natus Medical Inc., Middleton, WI, USA). All individuals included in the normative database were reported to have no current or past diagnosis of injury affecting balance, not to be taking medications affecting the central nervous system or known to affect balance or coordination, to have no symptoms of dizziness or lightheadedness, no diagnosis of vestibular or neurological disorders, no psychological disorders, no history of two or more unexplained falls within the past 6 months, and normal vision with or without glasses. This dataset provides ranges in 10-year intervals. The manufacturer suggested testing procedures account for height in the standardization of the foot placement on the force plate as the patient is set up for testing.

### 2.2. Outcome Measures

Falls were recorded as part of the adverse events for the monitoring of the original exercise study. Specifically, a fall was identified as an unplanned occurrence when the body unintentionally came to rest on the ground or floor. No fractures or injuries requiring hospitalization or withdrawal from the study occurred as a result of falls. To prevent recall bias issues, fall reports were tallied throughout the 12-week study period by study staff via weekly phone calls. Subjects provided detailed context of the falls, which included the following examples: gardening, shoveling snow, retrieving items from a car, getting out of bed, tripping over an obstacle while walking, catching the toe while walking, turning while walking, and attempting to step up onto a curb. Participants performed the following tests: Timed Up and Go (TUG), Adult Myopathy Assessment Tool (AMAT), NeuroCom balance tests including the Modified Clinical Test of Sensory Interaction on Balance (mCTSIB) and the Motor Control Test (MCT), and strength tests of Maximum Voluntary Isometric Contraction (MVIC). Blood was collected to measure creatinine levels.

The TUG was performed once, and the time to complete the test was recorded. Participants began the test seated in a standard armchair against the wall and were instructed to stand up, walk 3 m, turn around, return to the chair, and sit back down at a safe speed [20,21]. Participants were allowed to use a gait aid, for safety reasons, if typically relied on for ambulation, and the chair armrests during the test.

The AMAT was performed once and was scored for the total score of 45, the functional subscale score of 21, and the endurance subscale score of 24. The test required participants to complete seven functional tasks (supine-to-prone, modified push-up, sit-up, supine-to-sit, arm raise, sit-to-stand, and step-up) and six endurance tasks (sustained head elevation, repeated modified push-ups, sustained arm raise, sustained hip flexion, sustained knee extension, and repeated heel raises). The test was administered and scored by a clinician experienced with the assessment [19].

The Modified Clinical Test of Sensory Interaction on Balance (mCTSIB) is a test of quiet stance that measures the use of sensory information (visual, vestibular, and somatosensory) for balance [22]. Participants stand quietly and completely still three times, for 10 s, under four different conditions: on a firm surface with eyes open, on a firm surface with eyes closed, on a foam surface with eyes open, and on a foam surface with eyes closed. The outcome measure of this test is the sway velocity of the center of gravity (degrees/second). Higher scores indicate worse balance. The average of three trials was analyzed. All balance testing was completed barefoot on a NeuroCom Balance SMART Equitest^®^ System (previously Natus Medical Inc. Middleton, WI, USA).

The MCT assesses the automatic postural response of a subject to unexpected external perturbations [22]. Participants stand on two movable force plates, which generate sudden translations of small (0.5 inch), medium (1 inch), and large (1.5 inches) magnitudes in the forward and backward directions. We only analyzed large backward translations to mimic a forward stumble. Participants stand as steady as possible with one foot on each force plate and their weight evenly distributed. The first outcome measure of this test is the latency of their automatic response, defined as the time-lapse, in milliseconds, between the onset of the force plate translation and the automatic active force response of the subject, measured individually for each lower limb [22]. Higher scores indicate slower responses. The second outcome measure was the strength of plantar flexion response (amplitude scaling), a unitless value indicating the strength of the participant’s response in relation to each perturbation [22]. Abnormally high amplitude scaling demonstrates an overcorrection, and abnormally low demonstrates an undercorrection or possible motor weakness. Three trials were averaged for each leg. Any loss of balance or compensatory strategy by the subject was noted, and the trials were interrupted.

Maximal Voluntary Isometric Contraction (MVIC) was recorded using the fixed-frame dynamometry system Quantitative Muscle Assessment (QMA) (Aeverl Medical, Flowery Branch, GA, USA) [13]. The muscle groups included in this analysis were hip extensors, knee extensors, ankle dorsiflexors, and ankle plantar flexors, measured bilaterally. Participants were instructed to exert their maximum force for two trials lasting 5 s each, and the average of the two trials was utilized. Strength was recorded in kilograms, and the percentage of predicted strength was calculated according to previously published normative equations that account for sex, age, height, weight, muscle group, and body side [23]. Fixed-frame MVIC of ankle plantarflexion does not have a reliable normative equation; therefore, the percentage of predicted strength was calculated by comparing strength to mean control ankle plantarflexion (40 kg) in unpublished data previously measured in our lab using identical techniques and the same examiner (10 healthy men, mean age 51 ± 6 years, mean BMI 27.0 ± 4, standard error of the measurement 3.7 kg, intra-rater intraclass correlation coefficient 0.82, and inter-rater intraclass correlation coefficient 0.85).

Serum creatinine was measured from blood collected and analyzed by the NIH Clinical Center Department of Laboratory Medicine at the time of the baseline visit. Patients enrolled in this study did not have comorbidities or cognitive impairments or use medications that would have placed them at increased risk of falls.

### 2.3. Statistical Analysis

Falls were used as a dichotomous variable. Individuals who reported at least one fall during the 12-week study period were classified as “fallers”, and those with no falls were classified as “non-fallers”. After checking for data normality, the two groups were compared for age, disease duration, strength, balance, TUG, AMAT, and creatinine levels. Independent sample *t*-tests were used for parametric data and Wilcoxon rank tests for nonparametric data. Additionally, 95% confidence intervals (95% CI) and effect sizes were calculated for each comparison (effect sizes reported as Phi (*^φ^*) for the chi-square test, Cohen’s *D* for parametric data, and as correlation coefficient r for non-parametric data). The following criteria were used when interpreting effect sizes for Cohen’s *D*: small (0.20), moderate (0.50), and large (0.80); for coefficient *r:* small (0.10), moderate (0.30), and large (0.50); and for *^φ^:* small (0.10), moderate (0.30), and large (0.50) [24]. Significance was established at 0.05. Analyses were performed with R software (R Foundation for Statistical Computing, version 3.6.0, Vienna, Austria). Variables found to be significantly different between groups received post hoc receiver operating characteristic (ROC) curve analysis (Bendix Carstensen, Martyn Plummer, Esa Laara, and Michael Hills [2022]. Epi: A Package for Statistical Analysis in Epidemiology. R package version 2.47.1.) We interpreted the data as follows: an area under the curve (AUC) of 0.5 suggests no discrimination, 0.6–0.7 is considered poor, 0.7–0.8 acceptable, 0.8–0.9 excellent, and more than 0.9 outstanding [25]. Sensitivity and specificity were also calculated post hoc to establish optimal cutpoints for the significant variables. In addition, a power calculation was run on the variables with acceptable AUC values.

## 3. Results

Fifty men with SBMA were included in the analysis. Thirty-six percent (18 subjects) reported at least one fall over the 12-week study period; 8 subjects fell once, 9 fell twice, and 1 fell three times. The average age of the entire cohort was 55 ± 9 years. Of the 50 men, 58% reported relying on a gait aid. CAG repeat length ranged from 41 to 68, with an average of 47 ± 3.9 repeats. BMI ranged from 19.1 to 41.8, with an average of 28.2 ± 4.7.

Table 1 shows the comparison between fallers and non-fallers on each variable measured, as well as effect sizes. The groups were similar in age, disease duration, and BMI. There was a difference in gait aid use between fallers and non-fallers (*p* = 0.01).

Fallers were significantly weaker than non-fallers at the R ankle PF MVIC (*p* = 0.03) with a moderate effect size (*d* = 0.61). Also, fallers performed the TUG test significantly more slowly (*p* = 0.02) with a moderate effect size (*r* = 0.34), and their endurance performance on the AMAT was lower than non-fallers (*p* = 0.01) with a moderate effect size (r = 0.38). Measures of L ankle PF (*p* = 0.10, *d* = 0.48) and total LE strength (*p* = 0.07, *d* = 0.51) approached significance.

**Figure 1:**
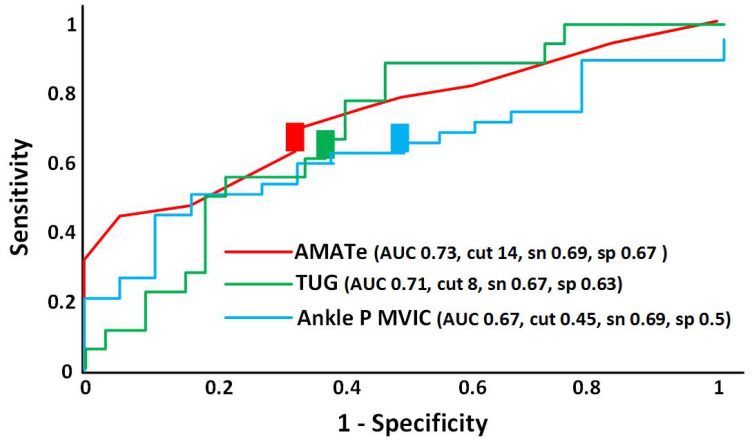
Receiver operating characteristics (ROC) curve for the Adult Myopathy Assessment Tool endurance subscale (AMATe—red line), Timed Up and Go Test (TUG—green line), and Ankle Plantarflexor Maximal Voluntary Isometric Contraction (Ankle P MVIC—blue line). Colored rectangles represent the cutoff points for each measure. Note: AUC = area under the curve; cut = cut point of optimized sensitivity and specificity; sen = sensitivity; spec = specificity.

All three significant clinical variables had similar discriminatory value in the high 0.60 s or low 0.70 s, which are considered acceptable according to published criteria (Figure 1). A cutoff value of 14 points for the endurance score on the AMAT yielded the best optimization of sensitivity (0.69) and specificity (0.67). A cutoff value of 8 s on the TUG had the best optimization of sensitivity (0.67) and specificity (0.63). A cutoff value of 45% of predicted right ankle PF strength optimized sensitivity (0.69) and specificity (0.50). Power was 83% for the AMAT endurance subscale score, 53% for TUG, and 58% for ankle PF strength.

## 4. Discussion

Falling, in those with SBMA, is common and is viewed as a dangerous disease consequence. Research examining the incidence of falls in people with SBMA is lacking. In the present study, 36% of people with SBMA reported at least one fall during the 12-week study period. Importantly, inclusion criteria required being ambulatory for community distances, indicating a relatively high rate of falling over a short time in people with moderate function. Fall incidence in this group of patients was higher than that reported for a variety of neuromuscular disorders (27%) in a prospective study over a similar 3-month period [26], which highlights the clinical relevance of falls in SBMA. Disease severity likely explains the high fall rate observed among this cohort, as these patients demonstrated considerable deficits in muscle strength and balance and often used assistive devices, which together make them susceptible to falling [15]. Three clinical measures discriminated fallers from non-fallers, and each had a moderate effect size. The TUG test time (longer time), the AMAT endurance subscale score (lower score), and ankle plantarflexion percentage of predicted MVIC values (weaker) showed acceptable clinical utility for predicting future falls in people with SBMA. However, no one clinical test should be used in isolation to screen for fall risk and clinicians are encouraged to use clinical judgment to determine the need for comprehensive fall assessment.

### 4.1. The Utility of the TUG Test to Identify Those at Risk for Falling

The TUG test is commonly used as a fall prediction test in various neuromuscular or geriatric populations [21,27,28,29,30]. Those who fell during this 12-week trial took longer compared with non-fallers to complete the TUG with a mean value (11.78 ± 5.65) similar to fall risk time thresholds seen in patients with Parkinson’s Disease (>11.5 s) [29], community-dwelling older adults (>13.5 s) [27], and patients with ALS (>14 s) [20]. A TUG threshold of >8 s was chosen to optimize sensitivity (66%) and specificity (63%). This implies that a TUG time above 8 s will accurately predict future fallers two-thirds of the time and will falsely predict fallers 37% of the time. Of note, this 8 s TUG threshold time is also the mean value for healthy men aged 60–69 [30].

The TUG test allows participants to use upper extremity support on the chair armrests as well as their “usual” gait aid to complete the test. In this cohort, 59% of patients used a gait aid (14 out of 32 non-fallers and 15 out of 18 fallers). Given the ease of TUG test administration, we suggest the utility of the >8 s TUG for predicting future fallers with SBMA is high; however, this needs to be confirmed given its low power for our sample size. Additionally, the finding of a higher percentage of fallers using a gait aid during the TUG test points to a possible confounding effect. One could argue, though, that patients who used a gait aid during TUG testing received a benefit for safety and stability, which could have improved their test time.

Some authors have pointed out the limitations of TUG for discriminating fallers from non-fallers in healthy, high-functioning older people but note more discriminatory values in less healthy, lower-functioning older people [31]. They suggest that “quick, multifactorial fall risk screens” should be considered over the TUG test. In addition to TUG testing, we support the importance of well-validated, performance-based, multifactorial fall risk screens for patients with SBMA in future trials.

### 4.2. The Adult Myopathy Assessment Tool Endurance Subscale Can Also Detect Fall Risk in Those with SBMA

The AMAT is a validated tool used in SBMA research to detect the rate of disease progression and correlates with composite MVIC, timed 2 min walk, ADL assessment, and SF36QOL PCS [0.82–0.91; *p* < 0.0001] [19]. In previous SBMA cohorts, the AMAT endurance subscale identified greater deficits (60% of predicted) compared with the AMAT functional subscale (70% of predicted) [18,19]. Impaired muscle endurance may represent an underappreciated aspect of muscle performance in SBMA that may contribute to falling. A recent study investigating exercise tolerance in SBMA revealed very short bouts of high-intensity cycling exercise were better tolerated and led to improved VO2max [32] compared with continuous cycling that was not well tolerated and did not result in VO2max improvements [33]. The authors suggested that neuronal fatigue may play a role, but more research is needed in this area. The AMAT endurance subscale requires sustained holding (90 s) of the limbs or head against gravity. While muscle weakness is the hallmark impairment of SBMA and MVIC receives much attention, the inability to sustain or repeat submaximal muscle contractions for relatively short periods of time may play an important role in falling. Using the same approach as above to optimize for sensitivity, we identified a fall risk cutoff score on the AMAT endurance subscale of ≤14 with a sensitivity of 69% and a specificity of 67%.

### 4.3. The Role of Weakness as a Risk Factor for Falling

It has been suggested that muscle weakness may be the most significant factor leading to falls [15]. Our results suggest that MVIC may not be as useful for predicting falls as we anticipated. Composite lower extremity MVIC percentage of predicted mean values (hip and knee extensors and ankle plantar flexors and dorsiflexors) approached significance (*p* = 0.07) for discriminating fallers (49%) from non-fallers (59%) with a moderate effect size (Table 1). However, when these four muscle groups were considered in isolation, only ankle plantarflexion strength helped predict falls (Table 1); however, this needs to be confirmed given its low power for our sample size.

Several studies have supported the relationship between weak ankle muscles and the incidence of falls and/or fear of falling [34,35,36,37,38], especially when ankle strength begins to fall below a functional threshold [39]. In our study, the threshold for fall risk was 45% of predicted ankle plantar flexor strength. Ankle muscles play an important role in preventing excessive postural sway and therefore maintaining balance [36], which may explain the significance of adequate ankle strength in preventing falls. A fall prevention program by Clemson et al. (2012) that focused on balance and lower limb strength training activities led to increased ankle strength and decreased falls in the intervention group compared to controls [39]. Authors have suggested that ankle-strengthening exercises be included in rehabilitation strategies to improve balance and prevent falls [40,41].

### 4.4. Unexpected Study Findings

Men with SBMA who sustained a fall were older, had a longer disease duration, and more often used a gait aid, but none of these variables reached statistical significance to discriminate fallers from non-fallers, except for gait aid use. Recent studies have shown a correlation of serum creatinine levels (proxy biomarker for whole-body lean muscle mass) with disease severity [42] and performance on the SBMA Functional Rating Scale [43]. Reductions in creatinine levels were detected longitudinally during the disease course [43,44]. In the present study, serum creatinine levels were considerably below reference ranges and were also lower in those who sustained a fall, but this also did not reach statistical significance. Balance and strength deficits are known to be risk factors for falls [15]. Surprisingly, the ROC analysis of balance measures did not discriminate between fallers and non-fallers. We suggest this is because the entire cohort has poor balance. For example, the normal sway velocity in our cohort’s age range is 1.60 degrees/second (standard deviation 0.50), whereas our fallers’ sway velocity was 3.98 and non-fallers’ was 3.35. We present an in-depth analysis of the aforementioned balance abnormalities for the same cohort elsewhere [45]. Additionally, our findings show postural adjustments in response to perturbations were also abnormally slow for both groups. These findings may indicate that sensory postural control and postural reactions are more severely affected than previously thought in patients with SBMA who are ambulatory in the community with and without assistive devices. Equally puzzling, strength differences in certain key lower extremity muscle groups, including hip and knee extensors, also did not discriminate between the groups, although both muscles trended toward being stronger in non-fallers. One possible explanation for this finding is the lack of data variance in these two key muscle groups. Hip extensors mean MVIC was relatively preserved in the entire cohort, with 88% and 102% of predicted strength in fallers and non-fallers, respectively. In this study, mean knee extensor muscle strength for fallers (31%) and non-fallers (41%) were not significantly different but indicated a severe impairment level that may have a considerable impact on fall risk [46,47]. Kwofie et al. assessed fall risk, via the Berg Balance Scale (BBS), in 16 healthy volunteers with a mean age of 29 years and found low fall risk with a mean score of 56 out of a possible 56. Sixty minutes after a femoral nerve block, knee extensor MVIC was reduced to 41% of baseline testing, and the mean BBS score declined to 37/56, indicating medium fall risk [48]. Given the similar profile of knee extensor weakness in our cohort, it is plausible that all subjects in this trial have increased fall risk due to knee extension muscle weakness.

### 4.5. Study Limitations and Future Directions/Applications

A limitation of this study is our small sample size, especially relating to fallers (18 subjects). It is important to remember, however, that this study was a post hoc analysis of a larger clinical trial, for which a power calculation resulted in a sample size of 50. It is possible that there would have been more fallers had the trial continued for longer than 12 weeks. This limitation did not affect the discriminative power for AMAT endurance to detect fallers, but it suggests further research is needed with larger sample sizes to address the low discriminative power for TUG and plantarflexion strength.

This study was also limited by a small control group for the ankle plantarflexion MVIC variable. We would have preferred to use a large normative database, but to our knowledge, none with similar methods exists. Nevertheless, the similar group demographics (control/cohort age: 51/55 and BMI: 27/28) and good statistical parameters (although unpublished) of our control group give us confidence in our findings. Additionally, we recognize that quantifying ankle plantar flexor strength currently requires fixed-frame dynamometer testing, which is time-consuming and expensive.

Early clinical efforts for strength preservation are advised, especially for the quadriceps and ankle plantar flexors, since these muscle groups are impaired in SBMA and implicated with fall risk. Since fall risk is multifactorial, we also suggest interventions to improve automatic balance reactions and sensory components of balance as well as to safely maintain or improve muscle endurance. Given the acceptable discriminatory value and adequate power of the AMAT endurance subscale score, this test may prove useful to assess disease progression in longitudinal studies as well as the effects of potential interventions.

## 5. Conclusions

This study provides evidence that three clinical measures can help detect fall risk in people with SBMA. Early detection of mobility (TUG > 8 s), muscle strength (ankle plantar flexion MVIC < 45% of predicted), and muscle endurance (AMAT endurance subscale < 14) impairments may help clinicians implement gait aid use or other fall prevention strategies before catastrophic falls occur.

## Figures and Tables

**Table 1:** Comparison of faller and non-faller outcomes.

Measure	Unit	Mean ± SD		*p*-Value	Effect Size	95% CI
		Fallers (*n* = 18)	Non-Fallers (*n* = 32)			
Demographics						
Age	years	58.0 ± 9.4	53.7 ± 8.6	0.12	−0.48 ^d^	[−1.15, 0.07]
Disease duration	years	18.7 ± 11.1	13.8 ± 7.6	0.11	0.23 ^r^	[0.02, 0.46]
Body mass index		27.7 ± 3.24	28.6 ± 5.42	0.48		
Gait aid use	%	83.3	43.8	**0.01** *	0.34 ^φ^	
Strength						
L Hip Ext	% predicted	88.5 ± 28.6	97.0 ± 30.9	0.34	0.28 ^r^	[−0.30, 0.90]
R Hip Ext	% predicted	88.2 ± 28.0	101.9 ± 41.0	0.37	0.13 ^r^	[−0.17, 0.89]
L Knee Ext	% predicted	31.7 ± 13.7	41.2 ± 25.1	0.25	0.17 ^r^	[0.01, 0.42]
R Knee Ext	% predicted	29.2 ± 11.9	41.3 ± 25.3	0.14	0.21 ^r^	[0.02, 0.45]
L Ankle DF	% predicted	34.4 ± 16.4	43.3 ± 20.3	0.14	0.21 ^r^	[0.02, 0.45]
R Ankle DF	% predicted	38.0 ± 14.8	43.9 ± 20.3	0.34	0.14 ^r^	[0.01, 0.40]
L Ankle PF	% predicted	41.9 ± 19.3	52.8 ± 25.8	0.10	0.48 ^d^	[−0.10, 1.10]
R Ankle PF	% predicted	42.0 ± 18.6	56.4 ± 27.7	**0.03** *	0.61 ^d^	[0.06, 1.20]
Total LE	% predicted	49.3 ± 14.1	58.8 ± 22.5	0.07	0.51 ^d^	[−0.01, 1.04]
Balance						
MCT LatBLR	ms	143.8 ± 13.3	144.8 ± 15.6	0.94	0.02 ^r^	[0.01, 0.38]
mCTSIB Foam Eyes Closed	deg/s	3.98 ± 1.85	3.35 ± 1.82	0.21	0.19 ^r^	[0.01, 0.44]
Function						
TUG	s	11.78 ± 5.65	9.15 ± 4.48	**0.02** *	0.34 ^r^	[0.09, 0.56]
AMAT functional	(max 21 pts)	13.94 ± 4.14	15.63 ± 3.23	0.15	0.45 ^d^	[−0.18, 1.22]
AMAT endurance	(max 24 pts)	12.28 ± 2.76	15.09 ± 3.53	**0.01** **	0.38 ^r^	[0.13, 0.60]
Biomarkers						
Creatinine	mg/dL	0.468 ± 0.15	0.531 ± 0.18	0.18	0.19 ^r^	[0.01, 0.46]

Notes: * *p* < 0.05, ** *p* < 0.01. Abbreviations: ^d^ = Cohen’s *D*, ^r^ = correlation coefficient r, *^φ^* = Phi, R = right, L = left, CI = confidence interval, % predicted = percent of predicted strength based on normative equation [23], Ext = extension, DF = dorsiflexion, PF = plantarflexion, LE = lower extremity, MCT LatBLR = latency (ms) of response to backward large right perturbation on Motor Control Test, mCTSIB foam eyes closed = sway velocity (deg/s) during foam surface/eyes closed condition on mCTSIB.

## Data Availability

The de-identified data used to support the findings of this study are available from Christopher Grunseich, christopher.grunseich@cc.nih.gov, for researchers who meet the criteria to access the data.
